# Life-history omnivory in the fairy shrimp *Branchinecta orientalis* (Branchiopoda: Anostraca)

**DOI:** 10.1007/s10750-022-05132-z

**Published:** 2023-01-17

**Authors:** Dunja Lukić, Navid Pormehr, Lynda Beladjal, Csaba F. Vad, Robert Ptacnik, Gilbert Van Stappen, Naser Agh, Zsόfia Horváth

**Affiliations:** 1 WasserCluster Lunz, Lunz am See, Austria; 2 Research Department for Limnology Mondsee, University of Innsbruck, Innsbruck, Austria; 3 Laboratory of Aquaculture & Artemia Reference Centre, Department of Animal Sciences and Aquatic Ecology, Faculty of Bioscience Engineering-Block F, Ghent University, Ghent, Belgium; 4 Terrestrial Ecology Unit, Department of Biology, Faculty of Science, Ghent University, Ghent, Belgium; 5 Institute of Aquatic Ecology, Centre for Ecological Research, Budapest, Hungary; 6 Laboratory of Aquatic Ecology, Evolution and Conservation, KU Leuven, Leuven, Belgium; 7 National Multidisciplinary Laboratory for Climate Change, Centre for Ecological Research, Budapest, Hungary; 8 Department of Biology and Aquaculture, Artemia and Aquaculture Research Institute, Urmia University, Urmia, Iran

**Keywords:** Juvenile anostracan, Nauplius, Ontogenetic omnivore, Planktonic prey, Aquatic food webs, Temporary ponds

## Abstract

Very little is known about the feeding of naupliar and juvenile life stages of omnivorous fairy shrimps (Crustacea: Anostraca). Here, we aim to reveal whether the fairy shrimp *Branchinecta orientalis* is an ontogenetic omnivore and at which age and ontogenetic stage they gain the ability to feed on zooplankton. We assess how food uptake rates change with age until reaching maturity by providing algae (pico- and nanoplanktonic unicellular algae) and zooplankton (rotifers and copepod nauplii) as food in individual experiments. We found that the fairy shrimp *B. orientalis* started to feed on both types of algal prey immediately after hatching. Nanoplanktonic algae likely represented the most important food source until reaching maturity. Moreover, fairy shrimps started to feed on zooplankton already when they were 7 days old. Slow-moving rotifers gradually gained importance in the fairy shrimp diet with time. Our results reveal an ontogenetic change in the prey spectrum of fairy shrimp. The systematic shift towards omnivory likely affects both phyto- and zooplankton community composition, possibly contributing to temporal changes in food web dynamics in fairy shrimp habitats, and temporary ponds, which may warrant more detailed investigations in future studies.

**Supplementary Information:**

The online version contains supplementary material available at 10.1007/s10750-022-05132-z.

## Introduction

Trophic omnivory, i.e., feeding on multiple trophic levels (Pimm & Lawton, 1978; Coll & Guershon, 2002) is a frequent component of all food webs (Holt & Polis, 1997; Thompson et al., 2007; Kratina et al., 2012). Omnivores can have critical roles in food webs by establishing numerous trophic interactions with other community members with different trophic positions (reviewed in Wootton, 2017). The feeding of an omnivore plays an important role in community structure and stability (Neutel et al., 2007; Stouffer & Bascompte, 2010; Wootton, 2017). Feeding habits of omnivorous organisms can vary over time in response to changes in the local environment as found for diverse aquatic systems [e.g., temperature (Boersma et al., 2016), productivity (Diehl & Feissel, 2000, 2001), turbidity (Lukić et al., 2020)]. Moreover, some animals are ontogenetic omnivores, meaning that they change feeding modes and habits with age, especially taxa that undergo metamorphosis, e.g., insects (Bernays, 1998), fish (Persson et al., 2000), or amphibians (Pimm & Rice, 1987). Thus, to have a realistic understanding of the roles of omnivores in food webs over their life cycle, it is of key importance to understand their feeding modes and diet spectrum across all life stages.

Temporary ponds are attractive model systems for ecological studies due to their small size and frequent occurrence along environmental gradients (De Meester et al., 2005). Even though they are relatively simple systems, they have a great ecological significance by hosting diverse and often endemic flora and fauna (Zacharias et al., 2007; EPCN, 2008; Vad et al., 2017). Fairy shrimps (Crustacea, Anostraca) are an endemic group of temporary ponds where they likely play an important role in the food webs with strong grazing impact (Yang & Park, 2017) and capacity for quick monopolization (Jocque et al., 2010). Although traditionally considered as filter feeders, feeding predominantly on phytoplankton and organic debris, some fairy shrimps, e.g., *Branchinecta orientalis* G.O. Sars 1901 (Lukić et al., 2018), *Streptocephalus proboscideus* (Frauenfeld, 1873) (Dumont et al., 1994), and *Chirocephalus diaphanus* Prevost 1803 (Sarma & Nandini, 2002) consume zooplankton as well. Thus, several fairy shrimps can be considered as intraguild predators of zooplankton, acting as both competitors and predators. Therefore, there is an increasing interest and need to understand and quantify the role of fairy shrimps in food webs and community structuring of temporary ponds.

A comprehensive understanding of the feeding biology and diet of fairy shrimps is still lacking, and available knowledge is mostly based on field and laboratory observations, with few evidence from laboratory experiments (reviewed in Lukić et al., 2018). Most fairy shrimps are predominantly non-selective filter feeders, feeding on algae, organic and inorganic detritus that they filter from the water column, or by mixing and scraping the bottom sediment (Brendonck, 1993; Paggi, 1996; Brendonck et al., 2008). Their diet is likely to broaden towards larger food items as they grow and according to the very few existing reports on this, ingestion rates are increasing with the size of adults within a given species (Daborn, 1975; Dumont & Ali, 2004; but see Lukić et al., 2018). For small- and medium-sized fairy shrimps (< 4 cm in adult length), there is only scattered information about the diet of adult individuals, while data on nauplii and juveniles are almost completely lacking (but see Dumont et al., 1994; Ali et al., 1996). Only a few larger species from the genus *Branchinecta* have been well documented to be predominantly predators, feeding on other crustaceans such as copepods and other fairy shrimps (White et al., 1969; Rogers et al., 2006; Rogers & Timms, 2017). This predatory feeding mode is reflected in the ontogenetic development of thoracopod morphology. Observations on large predatory fairy shrimp species such *Branchinecta ferox* (Milne Edwards, 1840) and *Branchinecta raptor* Rogers et al., 2006 showed that they gradually lose the ability of filter feeding with growth, possibly once they reach 4–5 cm in length (Fryer, 1983; Rogers et al., 2006).

Recent findings indicate that adult *B. orientalis* can ingest a wide range of prey types from pico-sized algae to rotifers and copepods, with no major differences in the prey-specific ingested biomass (Lukić et al., 2018). However, very few studies reported observations (e.g., in *B. ferox*; Fryer, 1983) or quantitative evidence (e.g., in *S. proboscideus*, Dumont et al., 1994; Ali et al., 1996) on the ingestion rates of naupliar and juvenile life stages of fairy shrimps. To date, no study compared the ingestion rates on different prey groups (phyto- *versus* zooplankton) commonly found in their natural habitats.

Our main aim here is to quantify the ontogenetic shift in food uptake of *B. orientalis*. To do so, we apply short-term laboratory feeding tests and measure age-specific biomass ingestion rates in *B. orientalis* (from nauplii to adults), by providing them various food types, including microalgae (pico- and nanoplanktonic unicellular algae) and zooplankton (rotifers and copepod nauplii). We hypothesize that they are initially herbivores and acquire the ability to feed on zooplankton during their ontogenetic development. Specifically, we aim to determine at what age they become omnivores (i.e., gain the ability to feed on zooplankton).

## Methods

### Stock culture of *B. orientalis*

Sediment containing *B. orientalis* resting eggs was collected in spring 2017 from the temporary soda pan Oberer Stinkersee in Austria (47° 48′ 49″ N, 16° 47′ 34″ E) and stored dry at 4°C for several months. We separated resting eggs using the sugar flotation method (Onbe, 1978; Marcus, 1990) and incubated them in a climate chamber, with a light regime 16:8 (light:dark) and temperature of 18°C to induce hatching. Artificial soda water (1 g l^−1^ NaHCO_3_ solution; conductivity of 1 mS cm^−1^) was used as a hatching and culturing medium. Upon hatching, the fairy shrimps were kept in a 3-l plastic container, fed ad libitum with a mixture of unicellular algae (*Cryptomonas* sp., *Scenedesmus* sp. and *Chlamydomonas* sp.). The fairy shrimps used in the experiments were returned to the stock culture after the experiment.

### Phyto- and zooplankton stock cultures for fairy shrimp feeding

To quantify feeding on phytoplankton, two different sized freshwater microalgae were used as food. The coccoid green alga *Mychonastes* sp. (spherical shape; diameter 2–3 μm) was used representing picoplankton and the green alga *Chlamydomonas* sp. (depressed ellipsoid; 9–20 μm length) as a larger unicellular food. To test carnivorous feeding, two zooplankton taxa of soda pans were used: nauplii of the copepod *Arctodiaptomus spinosus* (Daday, 1891) with a length of 0.20–0.32 mm (Alois Herzig pers. comm.), which were collected from the soda pan Oberer Stinkersee in Austria (47° 48′ 49″ N, 16° 47′ 34″ E) and the rotifer *Brachionus plicatilis* (Müller, 1786) with a length of 0.12–0.29 mm (Snell & Carrillo, 1984), which were commercially purchased (Aquacopa GmbH, Germany). Algal stock cultures were grown in WC medium (Guillard & Lorenzen, 1972) under the same light regime used for the *B. orientalis* stock cultures. Stock cultures of *A. spinosus* and *B. plicatilis* were separately kept in the laboratory, using the same light regime, medium and algal food as for the *B.orientalis* stock culture.

### Experimental design

To experimentally test ingestion rates on the different food items, fairy shrimps were moved to 40 ml round vials directly from the stock culture on day 2, 7, 14, 21, and 28 after hatching (the stock culture contained food; hence, fairy shrimps were not starved prior the experiments). In each vial, the fairy shrimps were supplied with one of the four different prey types (two microalgae species; rotifers; copepod nauplii) with 3 replicates for each. Experiments were conducted under the same conductivity, light regime, and temperature as for the stock cultures. We applied triplicates of controls (i.e., without *B. orientalis*) for each prey type in parallel. For algal food, the initial algal biomass of 1 mg C l^−1^ was set in all phytoplankton vials. This concentration is 5× higher than the saturating food concentration reported for *Daphnia magna* Straus 1820 (Porter et al., 1982), to avoid food depletion throughout the experiment. The initial number of copepod nauplii or rotifers corresponded to natural densities of the zooplankton observed in the Austrian soda pans (Horváth et al., 2014), i.e., 10 copepod nauplii (i.e., 250 ind l^−1^) or 50 rotifers per vial (1250 ind l^−1^).

In all treatments, the number of *B. orientalis* was adjusted on each test day as a function of their size, corresponding to their age, i.e., four specimens per vial were used on day 2, three specimens on day 7, and one specimen on days 14, 21, and 28. The feeding experiments ran for one hour, after which algal cells and zooplankters were counted in all vials for subsequent calculations of ingestion rates. The medium in each vial containing algae was gently stirred prior to sampling 1 ml of the medium for algal density estimation, to prevent erroneous assessment due to algal sedimentation. For estimating zooplankton densities in the experimental vials at the end of the experiments, the entire medium in vials was checked and all remaining zooplankton individuals were counted. The feeding experiments ended on day 28 as *B. orientalis* did not continue to grow remarkably past this age.

### Calculation of biomass of food organisms and of biomass ingestion rates

The biovolume of algal food was calculated by measuring cellular dimensions and approximating them to simple geometrical bodies (sphere for *Mychonastes* and depressed ellipsoid for *Chlamydomonas*). Then, we calculated cell dry weight using the approximation that 40% of cell dry weight is carbon, and carbon biomass is 14% of cell biovolume (Bowie et al., 1985; Vadstein et al., 1988). For zooplankton biomass, we used an approximation of individual dry weight of 0.16 µg for *B. plicatilis* (Theilacker & McMaster, 1971) and 1.45 µg for *A. spinosus* nauplii (Alois Herzig pers. comm., based on earlier measurements Herzig, 1974).

Biomass (i.e., dry weight) ingestion rate per individual fairy shrimp was calculated using the equation for food abundance (Frost, 1972; Marin et al., 1986):

$$M = \frac{{gC_{0} Vm}}{N},$$

when *M* is the ingested biomass per individual and time (in μg ind^−1^ h^−1^); g, the grazing coefficient; *C*_0_, the initial density of phytoplankton cells (in cells ml^−1^) or of zooplankton (in ind ml^−1^) offered as food at the beginning of the experiment; *V* volume of medium (in ml); *m* average biomass (in μg) per phytoplankton cell or zooplankton individual; and *N* number of *B. orientalis* per vial.

The grazing coefficient (*g*) was determined according to the following formula (Marin et al., 1986):

$$g = k - \frac{{{\text{ln}} \left( {C_{t} } \right) - {\text{ln }}\left( {C_{0} } \right) }}{t},$$

where *k* the growth rate, depends on the change of algal concentration in the controls (applicable for phytoplankton); *C*_0_, initial concentration of phytoplankton cells (in cells ml^−1^) or of zooplankton (in ind ml^−1^) offered as food at the beginning of the experiment; *C*_*t*_, final concentration of phytoplankton cells or final concentration of zooplankton offered as food at the end of the experiment; and *t* duration of the experiment (in h).

### Statistical analysis

To test the relationships between biomass ingestion rates of *B. orientalis* against age, we used linear models (LMs) in *Mychonastes* and *A. spinosus* prey and generalized additive models (GAMs; available in ‘mgcv’ package; (Wood, 2011) in *Chlamydomonas* and *B. plicatilis* prey based on comparisons of LM and GAM models with the corrected Akaike information criterion (AICc; available in ‘MuMIn’ package; Barton, 2020) in each of the four cases. We chose 4 as a smooth term for the ingestion of *Chlamydomonas* and 3 for the ingestion of *B. plicatilis* based on comparisons of model fit with AICc. We used quasibinomial distribution to fit the relationship between ingestion rates on *B. plicatilis* against age (after standardizing the ingestion rates from 0 to 1) as this method provided better fit given the absence of feeding on rotifers at day 2 and 7. *P* < 0.05 was chosen as the threshold for statistical significance. All data were analyzed in R (R Core Team, 2020).

## Results

**Fig. 1:**
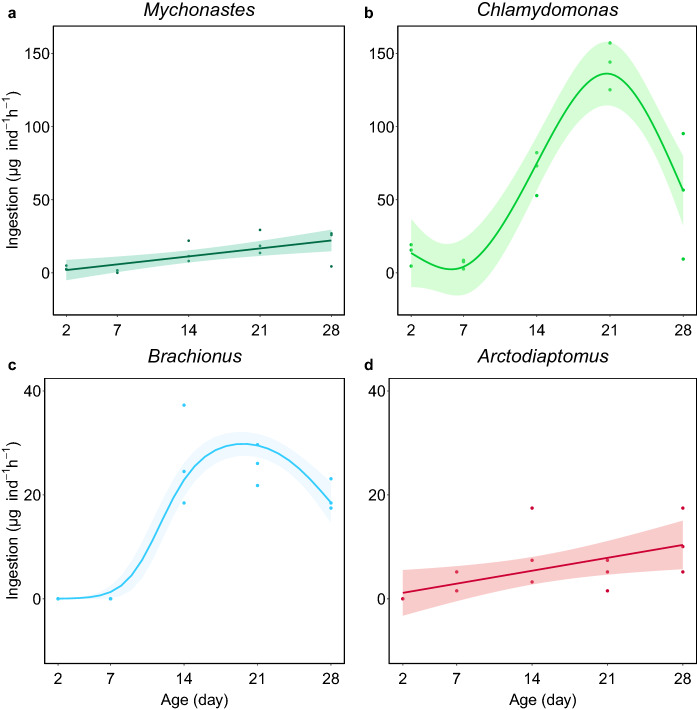
Biomass ingestion rates of *Branchinecta orientalis* (in dry weight; µg ind^−1^ h^−1^) on four different prey types during its growth and ontogenetic development: **a** picoplanktonic algae *Mychonastes* sp.; **b** nanoplanktonic algae *Chlamydomonas* sp.; **c** rotifer *Brachionus plicatilis* (the fit was generated with standardized values) and **d** nauplii of copepod *Arctodiaptomus spinosus*. Note the different scales of the y-axes for the algal and animal prey. *Branchinecta orientalis* developmental stages: day 2—(meta)nauplii; day 7—juveniles; day 14—larger juveniles or pre-adults, i.e., individuals morphologically like adults but females without visible ovisacs and males without developed clasping antennae; day 21—potentially reproductive individuals, i.e., distinguishable males and females; day 28—adults, individuals larger than on day 21

The first feeding test on day 2 showed that *B. orientalis* ingested both the picoalga *Mychonastes* and the larger alga *Chlamydomonas* from their early ages (Table S1). In contrast, feeding on zooplankton was first observed on day 7 with *A. spinosus* nauplii and day 14 with *B. plicatilis*. Over time, we found significant changes in the ingested biomass of *Mychonastes* (LM; *F*_df = 13_ = 14.18; *P* = 0.002; Fig. 1a), *Chlamydomonas* (GAM; *F*_ref. df = 2.99_ = 25.42; *P* < 0.001; Fig. 1b), *B. plicatilis* (GAM; *F*_ref. df = 1.99_ = 7.47; *P* = 0.008); Fig. 1c) and *A. spinosus* (LM; *F*_df = 13_ = 7.34; *P* = 0.018; Fig. 1d), showing that ingestion rates on all food types increased with the age of *B. orientalis*. From day 14, ingestion of *Chlamydomonas* was higher than all other food types (Fig. 1). When looking at zooplankton consumption, we observed higher ingestion rates on *B. plicatilis* compared to the nauplii of *A. spinosus*.

## Discussion

The findings show that *B. orientalis* is a life-history omnivore, feeding almost exclusively on pico- and nano-sized prey (e.g., planktonic algae) in its early life stages, while larger prey like zooplankton gradually gains importance later. Food web and feeding ecological studies typically focus on adult individuals, which might lead to a biased representation of the trophic role of life-history omnivores. Here, by studying the feeding of an invertebrate predator of temporary waters throughout its whole life history, we showed that *B. orientalis* was able to ingest a variety of phyto- and zooplankton species, different in size, trophic level, and locomotion already as a juvenile. From day 14, juvenile *B. orientalis* fed on both zooplankton prey types offered, rotifers and copepods, so its diet became increasingly like adult individuals (> 28 days old; Lukić et al., 2018, 2020).

We tested the feeding of *B. orientali*s on a broad range of prey during its larval and juvenile development to detect when they become omnivores (i.e., start feeding on zooplankton in addition to smaller prey groups as phytoplankton) and to quantify their overall impact on diverse groups of plankton. Our results on ingestion rates showed that *B. orientalis* nauplii and first-week juveniles are feeding almost exclusively on algae. Other pico-sized prey groups like bacteria and organic detritus were not tested in our experiments, but it is likely that these prey items are also important diet components of juvenile *B. orientalis* in its natural environment. In the first-week juveniles, ingestion rates on the different sized algae, *Mychonastes* and *Chlamydomonas*, were also similar. In later juvenile stages, *Chlamydomonas* (i.e., a larger unicellular algae) was ingested at higher rates than the smaller sized algae, with the highest rates across all other food items. The ingestion rates of juvenile *B. orientalis* on both algae were comparable to adult conspecifics and our results also imply that they maintain their feeding ability on pico- and nano-sized algae throughout their life (Lukić et al., 2018, 2020). However, nano-sized planktonic algae are probably their dominant prey items within phytoplankton likely due to mechanical limitations of their filtering apparatus, which could also be the case with other juvenile and adult anostracan species as it has been shown for *Artemia* (Bemal & Anil, 2019).

*Branchinecta orientalis* showed higher feeding rates on phytoplankton over zooplankton until 28 days old (i.e., when they reach maturity) but quickly started to feed on zooplankton as well (day 7). This represents one of the first records on carnivorous feeding in juvenile fairy shrimps (but see feeding of *S. proboscideus* in Dumont & Ali 2004). Juvenile *B. orientalis* fed slightly more efficiently on *B. plicatilis* prey than on the faster and somewhat larger calanoid nauplii, implying that other traits than size per se, such as swimming speed, escape response (i.e., jumping movements), and other motility features, may play an important role in fairy shrimp feeding efficiency. This was also found in adult *B. orientalis* (Lukić et al., 2018) and other fairy shrimps (Ali et al., 1996). Copepod nauplii avoid predation by jumping movements (Titelman & Kiørboe, 2003), which is likely the mechanism behind the observed slightly lower ingestion rates by *B. orientalis* compared to the similar sized *B. plicatilis* rotifers (that swim slowly and cannot jump; Buskey et al., 1993).

This study also shows that fairy shrimps might have a strong grazing impact on planktonic communities (e.g., exploitation competition with cladocerans; Jocque et al., 2010) already as juveniles. This finding slightly modifies the assumption of Lukić et al. (2018) that strong grazing pressure might start only later (around 1 month after rewetting and subsequent emergence of fairy shrimps), giving time for planktonic communities to establish high population size in a relatively predator-free community. Given that the population densities of juvenile fairy shrimps are much higher than the surviving adult population (see e.g., Horváth & Vad, 2015), the overall impact of fairy shrimps on other community members might be comparable during the presence of their local populations. In addition to their strong grazing pressure already in earlier life stages (around 2 weeks post hatching), our data also suggest stronger grazing pressure on the larger size class of phytoplankton (15–20 µm) over picoplankton (up to 2–3 µm in diameter). Under natural conditions, this could lead to indirect effects on zooplankton abundances through strong competition effects, while the selective grazing pressure on larger size classes within phytoplankton might contribute to an increased growth of picoplanktonic organisms (Leitão et al., 2018), which are the most dominant producers in several soda pans hosting fairy shrimps (Vörös et al., 2005; Felföldi et al., 2009; Somogyi et al., 2009). Thus, fairy shrimps might play an important role in shaping pelagic pond communities already shortly after inundation.

Our study provides the first experimental data on the feeding ecology of naupliar fairy shrimps, one of the key omnivorous components of the temporary pond food webs. Our results imply, based on the results of the example species *B. orientalis* that omnivorous fairy shrimps with their broad feeding spectrum likely have high grazing, predation, and competition impacts on the plankton communities of ponds. It is important to note that this study refers to the early inundation stages of temporary pond systems, i.e., at the time of early successional stages of communities and food webs, given that fairy shrimps are among the first to emerge from the local egg bank (Lukić et al., 2016) and many of them only have one generation per year (Brendonck, 1996). The ongoing climate change will likely decrease the inundation periods of ponds in temperate areas (Zacharias & Zamparas, 2010; Tuytens et al., 2014), which will lead to changes in species interactions. Therefore, the relative importance of fairy shrimps in temporary ponds may increase over time in parallel to shortening hydroperiod.

## Supplementary Information

Below is the link to the electronic supplementary material.

Supplementary file1 (DOCX 21 kb)

## Data Availability

The data generated during the current study are available in the supplementary material.

## References

[CR1] Ali AJ, Sarma SSS, Murugan G, Dumont HJ (1996). Effect of zooplankton type and abundance on prey consumption by the fairy shrimp, *Streptocephalus proboscideus* (Anostraca: Crustacea). Hydrobiologia.

[CR2] Barton, K., 2020. MuMIn: Multi-Model Inference. R package version 1.43.17., https://CRAN.R-project.org/package=MuMIn.

[CR3] Bemal S, Anil AC (2019). Picophytoplankton *Synechococcus* as food for nauplii of *Amphibalanus amphitrite* and *Artemia salina*. Hydrobiologia.

[CR4] Bernays EA (1998). Evolution of feeding behavior in insect herbivores. BioScience.

[CR5] Boersma M, Mathew KA, Niehoff B, Schoo KL, Franco-Santos RM, Meunier CL (2016). Temperature driven changes in the diet preference of omnivorous copepods: no more meat when it’s hot?. Ecology Letters.

[CR6] Bowie, G. L., W. B. Mills, D. B. Porcella, C. C. Campbell, J. R. Pagenkopf, G. L. Rupp, K. M. Johnson, R. W. H. Chan, S. A. Gherini, & C. E. Chamberlin, 1985. Rates, constants and kinetics formulations in surface water quality modeling, 2nd edn. Environment Research Laboratory, Environmental Protection Authority EPA/600/3-85/040.

[CR7] Brendonck L (1993). Feeding in the fairy shrimp *Streptocephalus proboscideus* (Frauenfeld) (Branchiopoda: Anostraca). I. Aspects of the feeding biology. Journal of Crustacean Biology.

[CR8] Brendonck L (1996). Diapause, quiescence, hatching requirements: what we can learn from large freshwater branchiopods (Crustacea: Branchiopoda: Anostraca, Notostraca, Conchostraca). Hydrobiologia.

[CR9] Brendonck L, Rogers DC, Olesen J, Weeks S, Hoeh WR (2008). Global diversity of large branchiopods (Crustacea: Branchiopoda) in freshwater. Hydrobiologia.

[CR10] Buskey EJ, Coulter C, Strom S (1993). Locomotory patterns of microzooplankton: potential effects on food selectivity of larval fish. Bulletin of Marine Science.

[CR11] Coll M, Guershon M (2002). Omnivory in terrestrial arthropods: mixing plant and prey diets. Annual Review of Entomology.

[CR12] Daborn G (1975). Life history and energy relations of giant fairy shrimp *Branchinecta gigas* Lynch 1937 (Crustacea-Anostraca). Ecology.

[CR13] De Meester L, Declerck S, Stoks R, Louette G, Van De Meutter F, De Bie T, Michels E, Brendonck L (2005). Ponds and pools as model systems in conservation biology, ecology and evolutionary biology. Aquatic Conservation: Marine and Freshwater Ecosystems.

[CR14] Diehl S, Feissel M (2000). Effects of enrichment on three-level food chains with omnivory. American Naturalist.

[CR15] Diehl S, Feissel M (2001). Intraguild prey suffer from enrichment of their resources: a microcosm experiment with ciliates. Ecology.

[CR16] Dumont HJ, Ali AJ (2004). Stage-specific cannibalism and spontaneous cyst hatching in the freshwater fairy shrimp *Streptocephalus proboscideus* Frauenfeld. Hydrobiologia.

[CR17] Dumont HJ, Ali AJ, Sarma SSS, Mertens J (1994). Predatory filter-feeding in fairy shrimps: functional response of *Streptocephalus proboscideus* (Crustacea: Anostraca) fed *Anuraeopsis fissa* (Rotifera). Internationale Revue Der Gesamten Hydrobiologie Und Hydrographie.

[CR18] EPCN, 2008. The pond manifesto. European Pond Conservation Network.

[CR19] Felföldi T, Somogyi B, Márialigeti K, Vörös L (2009). Characterization of photoautotrophic picoplankton assemblages in turbid, alkaline lakes of the Carpathian Basin (Central Europe). Journal of Limnology.

[CR20] Frost BW (1972). Effects of size and concentration of food particles on the feeding behavior of the marine planktonic copepod *Calanus pacificus*. Limnology and Oceanography.

[CR21] Fryer G (1983). Functional ontogenetic changes in *Branchinecta ferox* (Milne-Edwards) (Crustacea, Anostraca). Philosophical Transactions of the Royal Society of London Series B-Biological Sciences.

[CR22] Guillard RRL, Lorenzen CJ (1972). Yellow-green algae with chlorophyllide C. Journal of Phycology.

[CR23] Herzig A (1974). Some population characteristics of planktonic crustaceans in Neusiedler see. Oecologia.

[CR24] Holt RD, Polis GA (1997). A theoretical framework for intraguild predation. American Naturalist.

[CR25] Horváth Z, Vad CF (2015). Life history and current distribution of the fairy shrimp *Chirocephalus carnuntanus* (Brauer, 1877) (Crustacea: Anostraca). North-Western Journal of Zoology.

[CR26] Horváth Z, Vad CF, Tóth A, Zsuga K, Boros E, Vörös L, Ptacnik R (2014). Opposing patterns of zooplankton diversity and functioning along a natural stress gradient: when the going gets tough, the tough get going. Oikos.

[CR27] Jocque M, Vanschoenwinkel B, Brendonck L (2010). Anostracan monopolisation of early successional phases in temporary waters?. Fundamental and Applied Limnology.

[CR28] Kratina P, LeCraw RM, Ingram T, Anholt BR (2012). Stability and persistence of food webs with omnivory: Is there a general pattern?. Ecosphere.

[CR29] Leitão E, Ger KA, Panosso R (2018). Selective grazing by a tropical copepod (*Notodiaptomus iheringi*) facilitates *Microcystis* dominance. Frontiers in Microbiology.

[CR30] Lukić D, Vad CF, Horváth Z (2016). Isolation by sugar flotation has no direct effect on the hatching success of zooplankton resting eggs. Journal of Limnology.

[CR31] Lukić D, Horváth Z, Vad CF, Ptacnik R (2018). Food spectrum of *Branchinecta orientalis*—are anostracans omnivorous top consumers of plankton in temporary waters?. Journal of Plankton Research.

[CR32] Lukić D, Ptacnik R, Vad CF, Pόda C, Horváth Z (2020). Environmental constraint of intraguild predation: inorganic turbidity modulates omnivory in fairy shrimps. Freshwater Biology.

[CR33] Marcus N (1990). Calanoid copepod, cladoceran, and rotifer eggs in sea-bottom sediments. Marine Biology.

[CR34] Marin V, Huntley ME, Frost B (1986). Measuring feeding rates of pelagic herbivores: analysis of experimental design and methods. Marine Biology.

[CR35] Neutel A-M, Heesterbeek JAP, van de Koppel J, Hoenderboom G, Vos A, Kaldeway C, Berendse F, de Ruiter PC (2007). Reconciling complexity with stability in naturally assembling food webs. Nature.

[CR36] Onbe T (1978). Sugar flotation method for sorting the resting eggs of marine cladocerans and copepods from sea-bottom sediment. Bulletin of the Japanese Society of Scientific Fisheries.

[CR37] Paggi JC (1996). Feeding ecology of *Branchinecta gaini* (Crustacea: Anostraca) in ponds of South Shetland Islands, Antarctica. Polar Biology.

[CR38] Persson L, Byström P, Wahlström E (2000). Cannibalism and competition in Eurasian perch: population dynamics of an ontogenetic omnivore. Ecology.

[CR39] Pimm SL, Lawton JH (1978). On feeding on more than one trophic level. Nature.

[CR40] Pimm SL, Rice JC (1987). The dynamics of multispecies, multi-life-stage models of aquatic food webs. Theoretical Population Biology.

[CR41] Porter KG, Gerritsen J, Orcutt JD (1982). The effect of food concentration on swimming patterns, feeding behavior, ingestion, assimilation, and respiration by *Daphnia*. Limnology and Oceanography.

[CR42] R Core Team, 2020. A Language and Environment of Statistical Computing (v. 4.0. 2) [Computer software]. R Foundation for Statistical Computing.

[CR43] Rogers DC, Timms BV (2017). Predatory morphology and behaviour in *Branchinella occidentalis* (Dakin, 1914) (Branchiopoda: Anostraca: Thamnocephalidae). Proceedings of the Linnean Society of New South Wales.

[CR44] Rogers DC, Quinney DL, Weaver J, Olesen J (2006). A new giant species of predatory fairy shrimp from Idaho, USA (Branchiopoda: Anostraca). Journal of Crustacean Biology.

[CR45] Sarma SSS, Nandini S (2002). Studies on functional response and prey selection using zooplankton in the anostracan *Chirocephalus diaphanus* Prevost, 1803. Hydrobiologia.

[CR46] Snell TW, Carrillo K (1984). Body size variation among strains of the rotifer *Brachionus plicatilis*. Aquaculture.

[CR47] Somogyi B, Felfoeldi T, Vanyovszki J, Agyi A, Marialigeti K, Vörös L (2009). Winter bloom of picoeukaryotes in Hungarian shallow turbid soda pans and the role of light and temperature. Aquatic Ecology.

[CR48] Stouffer DB, Bascompte J (2010). Understanding food-web persistence from local to global scales. Ecology Letters.

[CR49] Theilacker GH, McMaster MF (1971). Mass culture of the rotifer *Brachionus plicatilis* and its evaluation as a food for larval anchovies. Marine Biology.

[CR50] Thompson RM, Hemberg M, Starzomski BM, Shurin JB (2007). Trophic levels and trophic tangles: the prevalence of omnivory in real food webs. Ecology.

[CR51] Titelman J, Kiørboe T (2003). Predator avoidance by nauplii. Marine Ecology Progress Series.

[CR52] Tuytens K, Vanschoenwinkel B, Waterkeyn A, Brendonck L (2014). Predictions of climate change infer increased environmental harshness and altered connectivity in a cluster of temporary pools. Freshwater Biology.

[CR53] Vad CF, Péntek AL, Cozma NJ, Földi A, Tóth A, Tóth B, Böde NA, Móra A, Ptacnik R, Ács É, Zsuga K, Horváth Z (2017). Wartime scars or reservoirs of biodiversity? The value of bomb crater ponds in aquatic conservation. Biological Conservation.

[CR54] Vadstein O, Jensen A, Olsen Y, Reinertsen H (1988). Growth and phosphorus status of limnetic phytoplankton and bacteria. Limnology and Oceanography.

[CR55] Vörös L, Balogh KV, Boros E (2005). Picoplankton predominance in soda lakes. Hidrológiai Közlöny.

[CR56] White GE, Fabris G, Hartland-Rowe R (1969). The method of prey capture by *Branchinecta gigas* Lynch, 1937 (Anostraca). Crustaceana.

[CR57] Wood SN (2011). Fast stable restricted maximum likelihood and marginal likelihood estimation of semiparametric generalized linear models. Journal of the Royal Statistical Society: Series B (Statistical Methodology).

[CR58] Wootton KL (2017). Omnivory and stability in freshwater habitats: does theory match reality?. Freshwater Biology.

[CR59] Yang D, Park S (2017). Freshwater anostracan, *Branchinella kugenumaensis*, as a potential controlling consumer species on toxic cyanobacteria *Microcystis aeruginosa*. Aquatic Ecology.

[CR60] Zacharias I, Zamparas M (2010). Mediterranean temporary ponds. A disappearing ecosystem. Biodiversity and Conservation.

[CR61] Zacharias I, Dimitriou E, Dekker A, Dorsman E (2007). Overview of temporary ponds in the Mediterranean region: threats, management and conservation issues. Journal of Environmental Biology.

