# Supplementary material for: Life-history omnivory in the fairy shrimp Branchinecta orientalis (Branchiopoda: Anostraca)
Source: Hydrobiologia. 2023 Jan 17;850(4):901–9. doi: 10.1007/s10750-022-05132-z (PMC9905153; doi:10.1007/s10750-022-05132-z)
Supplement: Supplementary file 1 — Supplementary file1 (DOCX 21 kb) [file 10750_2022_5132_MOESM1_ESM.docx]

***Hydrobiologia***

**Life-history omnivory in the fairy shrimp *Branchinecta orientalis* (Branchiopoda: Anostraca)**

Dunja Lukić, Navid Pormehr, Lynda Beladjal, Csaba F. Vad, Robert Ptacnik, Gilbert Van Stappen, Naser Agh, Zsόfia Horváth

Correspondence:

Dunja Lukić

WasserCluster Lunz, Austria & Research Department for Limnology Mondsee, University of Innsbruck, Austria

dunjalkc@gmail.com

**Table S1.** Ingestion rates of *Branchinecta orientalis* on different prey types: the picoalga *Mychonastes* sp., the nanoalga *Chlamydomonas* sp., the rotifer *Brachionus plicatilis* and the nauplii of the copepod *Arctodiaptomus spinosus*. Ingestion rates are presented as the number of ingested algal cells or zooplankton individuals per individual *B. orientalis* per hour and in ingested biomass (i.e., dry weight) per individual *B. orientalis* per hour. Units are shown in brackets.

| Prey | Age (day) | Ingestion rate (cells or individuals ind^-1^ h^-1^) | Biomass ingestion rate (µg ind^-1^ h^-1^) |
| --- | --- | --- | --- |
| *Mychonastes* sp. | 2 | 469308.15 | 2.22 |
| *Mychonastes* sp. | 2 | 549945.52 | 2.60 |
| *Mychonastes* sp. | 2 | 1042319.35 | 4.93 |
| *Mychonastes* sp. | 7 | 0 | 0 |
| *Mychonastes* sp. | 7 | 319368.33 | 1.51 |
| *Mychonastes* sp. | 7 | 362736.98 | 1.71 |
| *Mychonastes* sp. | 14 | 1697768.80 | 8.03 |
| *Mychonastes* sp. | 14 | 4642253.91 | 21.95 |
| *Mychonastes* sp. | 14 | 2404116.16 | 11.37 |
| *Mychonastes* sp. | 21 | 3900341.21 | 18.44 |
| *Mychonastes* sp. | 21 | 2872625.71 | 13.58 |
| *Mychonastes* sp. | 21 | 6203995.52 | 29.33 |
| *Mychonastes* sp. | 28 | 922128.28 | 4.36 |
| *Mychonastes* sp. | 28 | 5701122.50 | 26.95 |
| *Mychonastes* sp. | 28 | 5470148.43 | 25.86 |
| *Chlamydomonas* sp. | 2 | 20941.48 | 4.61 |
| *Chlamydomonas* sp. | 2 | 70567.32 | 15.52 |
| *Chlamydomonas* sp. | 2 | 87215.91 | 19.19 |
| *Chlamydomonas* sp. | 7 | 38812.40 | 8.54 |
| *Chlamydomonas* sp. | 7 | 34411.02 | 7.57 |
| *Chlamydomonas* sp. | 7 | 11856.24 | 2.61 |
| *Chlamydomonas* sp. | 14 | 373594.48 | 82.19 |
| *Chlamydomonas* sp. | 14 | 240043.88 | 52.81 |
| *Chlamydomonas* sp. | 14 | 332577.03 | 73.17 |
| *Chlamydomonas* sp. | 21 | 569155.91 | 125.21 |
| *Chlamydomonas* sp. | 21 | 655256.84 | 144.16 |
| *Chlamydomonas* sp. | 21 | 714594.74 | 157.21 |
| *Chlamydomonas* sp. | 28 | 257318.39 | 56.61 |
| *Chlamydomonas* sp. | 28 | 432876.88 | 95.23 |
| *Chlamydomonas* sp. | 28 | 42686.19 | 9.39 |
| *Brachionus plicatilis* | 2 | 0 | 0 |
| *Brachionus plicatilis* | 2 | 0 | 0 |
| *Brachionus plicatilis* | 2 | 0 | 0 |
| *Brachionus plicatilis* | 7 | 0 | 0 |
| *Brachionus plicatilis* | 7 | 0 | 0 |
| *Brachionus plicatilis* | 7 | 0 | 0 |
| *Brachionus plicatilis* | 14 | 75.71 | 12.11 |
| *Brachionus plicatilis* | 14 | 56.97 | 9.11 |
| *Brachionus plicatilis* | 14 | 115.13 | 18.42 |
| *Brachionus plicatilis* | 21 | 80.47 | 12.87 |
| *Brachionus plicatilis* | 21 | 91.63 | 14.66 |
| *Brachionus plicatilis* | 21 | 67.35 | 10.78 |
| *Brachionus plicatilis* | 28 | 53.94 | 8.63 |
| *Brachionus plicatilis* | 28 | 56.97 | 9.11 |
| *Brachionus plicatilis* | 28 | 71.36 | 11.42 |
| *Arctodiaptomus spinosus* | 2 | 0 | 0 |
| *Arctodiaptomus spinosus* | 2 | 0 | 0 |
| *Arctodiaptomus spinosus* | 2 | 0 | 0 |
| *Arctodiaptomus spinosus* | 7 | 1.05 | 1.53 |
| *Arctodiaptomus spinosus* | 7 | 1.05 | 1.53 |
| *Arctodiaptomus spinosus* | 7 | 3.57 | 5.17 |
| *Arctodiaptomus spinosus* | 14 | 5.11 | 7.41 |
| *Arctodiaptomus spinosus* | 14 | 2.23 | 3.23 |
| *Arctodiaptomus spinosus* | 14 | 12.04 | 17.46 |
| *Arctodiaptomus spinosus* | 21 | 5.11 | 7.41 |
| *Arctodiaptomus spinosus* | 21 | 3.57 | 5.17 |
| *Arctodiaptomus spinosus* | 21 | 1.05 | 1.53 |
| *Arctodiaptomus spinosus* | 28 | 6.93 | 10.05 |
| *Arctodiaptomus spinosus* | 28 | 12.04 | 17.46 |
| *Arctodiaptomus spinosus* | 28 | 3.57 | 5.17 |
